# Traditional Chinese Medicine Yimucao Injection Combined with Western Medicine for Preventing Postpartum Hemorrhage after Cesarean Section: A Systematic Review and Meta-Analysis

**DOI:** 10.1155/2019/7475151

**Published:** 2019-04-09

**Authors:** Shichun Chen, Baocheng Xie, Hao Tian, Shaobo Ding, Chengyu Lu

**Affiliations:** ^1^Department of Pharmacology, Guangdong Medical University, Dongguan 523808, China; ^2^Department of Pharmacy, Dongguan People's Hospital, Dongguan, 523000, China; ^3^Guangdong Key Laboratory for Research and Development of Natural Drugs, Guangdong Medical University, 524023 Zhanjiang, China

## Abstract

**Objective:**

Yimucao injection combined with several contraction uterus drugs is in use for preventing postpartum hemorrhage after cesarean section. The present study is a meta-analysis comparing the efficacy and safety of these drugs.

**Methods:**

PubMed, Cochrane Library, Embase, the China National Knowledge Infrastructure (CNKI), the Chinese Biomedical Database (CBM), VIP, and Wanfang database were searched until June 2018. We selected RCTs of Yimucao injection combined with western medicine for preventing postpartum hemorrhage and study quality was assessed using the revised Cochrane risk of bias tool. Forty-eight RCTs are comprised of 7,330 participants.

**Results:**

The overall response rate of Yimucao injection combined with western medicine as a class (OR=4.19, 95%CI=2.83, 6.20,* P*<0.00001) was found to be significantly improved than western medicine alone. Yimucao injection combined with western medicine group could significantly reduce blood loss in intraoperative (SMD= -1.15, 95%CI= -1.43, -0.87,* P*<0.00001), compared with control group. The treatment group could significantly reduce postpartum blood loss within 2 hours (SMD= -1.73, 95%CI= -2.01, -1.46,* P*<0.00001) and had a significantly lower blood loss within 24 hours (SMD= -1.92, 95%CI= -2.21, -1.63,* P*<0.00001) than control group. Additionally, in terms of the safety, Yimucao injection group reduced the risk of adverse events in the course of prevention than the western medicine group.

**Conclusions:**

This study demonstrated that Yimucao injection combined with western medicine may be more effective for preventing postpartum hemorrhage after cesarean section. However, high-quality and large multicenter randomized clinical trials will be needed to prove the consequence in the further.

## 1. Introduction

Postpartum hemorrhage (PPH) is one of most common diseases of maternal death worldwide and severe morbidity during pregnancy [1]. Uterine atony, injury of birth canal, abnormal placenta, and dysfunction of blood coagulation were the leading cause of PPH in pregnant women with cesarean section, and the average blood loss during cesarean section is significantly higher than that during vaginal delivery [2, 3]. Currently, western medicine such as oxytocin [4], carboprost tromethamine [5], and misoprostol [6] can play an important role in addressing the issue of PPH during and after cesarean section.

Oxytocin is an effective method to prevent uterine atony and PPH during cesarean section, and it is generally regarded that oxytocin is the first-line drug proposed by the world health organization (WHO) and other international guidelines [7]. However, the main characteristic of oxytocin is that the effect of contractile uterus was quick and short duration during the treatment of PPH, and it could be discontinues to contract uterine in certain dosage range and results in some side effects, such as tachycardia, hypotension, and arrhythmia [8].

A large number of studies confirmed that Yimucao injection as a traditional Chinese medicine is more effective for clinical prevention of postpartum hemorrhage. The main active components of Yimucao injection were alkaloids, and it has uterine contraction effects [9]. In addition, Yimucao injection combined with western medicine (oxytocin, carboprost tromethamine, and misoprostol) can not only prevent PPH during cesarean section and improve the curative effect, but also decrease the rate of adverse events. Even though several studies assessing the effect of Yimucao injection combined with western medicine increased dramatically in the decade, it is still lack of comprehensive systematic review to guidance. Therefore, our study included 48 RCTs with a total of 7330 pregnant women with cesarean section in order to investigate the effect of western medicine (oxytocin, carboprost tromethamine, and misoprostol) alone or combined with Yimucao injection in women after cesarean section, and we also performed subgroup analyses in order to acquire high-quality evidence, which comprehensive systematic review to assess the efficacy and safety of Yimucao injection as adjuvant treatment for preventing PPH during and after cesarean section.

## 2. Methods

### 2.1. Search Strategy

We systematically searched Medical databases, including PubMed, Cochrane Library, Embase, the China National Knowledge Infrastructure (CNKI), the Chinese Biomedical Database (CBM), VIP database, and Wanfang for RCTs examining the effect of Yimucao injection combined with western medicine (oxytocin, carboprost tromethamine, and misoprostol) for preventing postpartum hemorrhage after cesarean section, from their inception until June 2018. The searched the terms of medical keywords: (1) “yimucao”, “yimucao injection”, “leonurus japonicus injection”connected with “OR”; (2) “cesarean section”, “postpartum hemorrhage”, “cesarean section”, “abdominal delivery” connected with “OR”; (3) “randomized controlled” or “Clinical Trials”. Then, the above search terms of (1), (2), and (3) were connected with “AND”. We manually searched all research studies that are the references of the original and review articles for possible related studies.

### 2.2. Study Selection

This systematic review included 48 clinical studies that met the following criteria: (1) studies reported patients with cesarean section, (2) studies compared the effectiveness and safety of Yimucao injection combined with western medicine, (3) studies selected as randomized controlled trials (RCTs), and (4) studies included clinical outcomes which the estimated blood loss.

### 2.3. Data Abstraction

We collected relevant information including: study characteristics (publication year and sample size), participant characteristic (average age and cesarean section), interventions (type of administration, dose, treatment protocol, and duration of treatment), and outcomes (intraoperative blood loss, blood loss within 2 hours, blood loss within 24 hours, and adverse events). We assessed the clinical efficacy and safety of Yimucao injection combined with western medicine for preventing PPH according to the guideline on the information of extraction.

### 2.4. Quality Assessment

For eligible studies, data were extracted independently by the two authors and carried out a quality assessment process according to the predefined inclusion criteria. Disagreements were resolved by consensus or discussion with a third author. The methodological quality assessed the risk of bias of RCTs by the Cochrane risk of bias tool. We considered random sequence generation, allocation concealment, blinding, incomplete outcome data, selective outcome reporting, and other potential sources of bias. The modified instrument removed the “Low risk” option and added “Unclear risk” and “High risk”.

### 2.5. Statistical Analysis

In this meta-analysis, RevMan 5.3 software provided by the Cochrane Collaboration was used to perform the data analysis. The analyses of dichotomous data were presented as the risk ratios (RR) or odds ratios (OR), and the continuous data were presented as mean difference (MD) or standardized mean difference (SMD) with 95% CI. All clinical studies were measured by the chi-square test, with the *I*^2^ test. If the *I*^2^ was less than 50%, we considered that the heterogeneity among studies was small, and we used the fixed effects model for data analysis. If heterogeneity was detected (*I*^2^ value >50%), we suggest severe heterogeneity between the studies by considering possible factors, such as the dose of medicine, treatment course, and disease type. If the heterogeneity was still significant, we chose the random effects model or used only qualitative descriptions.

## 3. Results

### 3.1. Search Results

**Figure 1:**
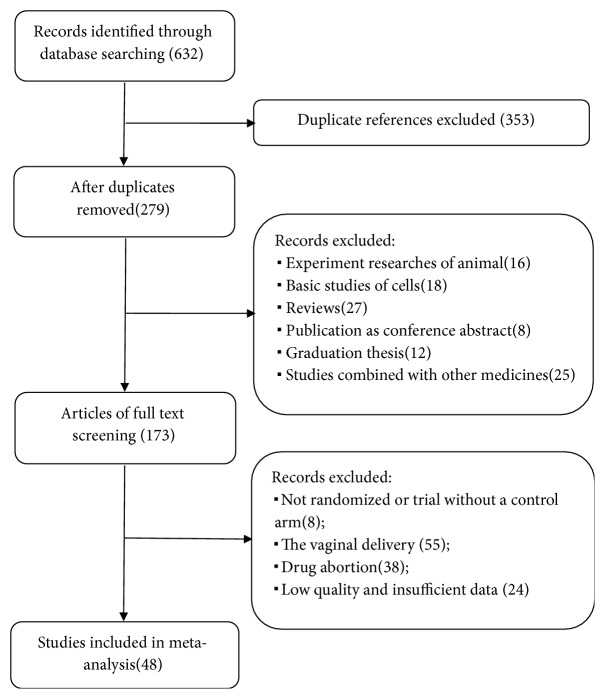
Flowchart and strategy of the meta-analysis.

Through the seven medical database searches, we found 632 citations from all searches and excluded 353 duplicates. After screening the titles and abstracts, we retrieved 279 full texts for further assessment. Of these, 231 were excluded for the following reasons: duplicate publication as conference abstract, animal experimentation, basic studies of cells, trial without a control arm, the treatment of Yimucao injection combined with other medicine, the vaginal delivery of study, graduation thesis, and narrative reviews; 48 trials involving 7330 women delivered were finally included (Figure 1).

### 3.2. Study Characteristics

In the 48 trials included, a total of 7330 pregnant women participated: the treatment group of Yimucao injection combined with oxytocin and the control group with oxytocin (32 studies); the treatment group of Yimucao injection combined with carboprost tromethamine and the control group with carboprost tromethamine (12 studies); the treatment group of Yimucao injection combined with misoprostol and the control group with misoprostol (4 studies). All the studies were conducted in China and were published in Chinese. Baseline characteristics were summarized in Table 1.

### 3.3. Quality Assessment

**Figure 2:**
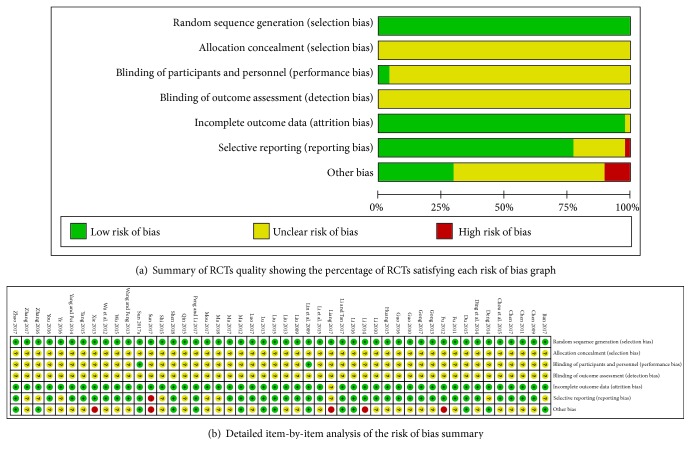


The methodological quality of included studies was estimated according to the bias risk assessment tools provided by the Cochrane Collaboration. All of the included trials mentioned randomized allocation and allocation concealment were unclear. Forty-six studies were at an unclear risk of bias for blinding of participants and personnel, 12 trials [10–21] described in detail the method of random number table, and only 2 trials [12, 19] described as double-blind. All trials reported methods with a low risk of incomplete outcome data and thirty-eight studies were at low risk of bias. These results are summarized in Figure 2.

### 3.4. Major Outcomes

#### 3.4.1. The Total Effective Rate

**Figure 3:**
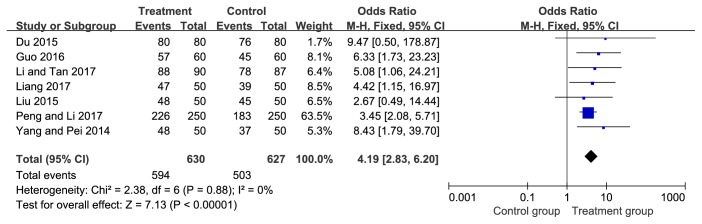
Forest plot of the meta-analysis with the total effective rate.

The total effective rate was reported in seven studies [10, 14, 17, 22–25] in which 630 patients in the treatment group and 627 patients in the control group. The meta-analysis was conducted, as shown in Figure 3, Yimucao injection combined with western medicine showed a better effect on the prevention of PPH compared with the western medicine alone (OR=4.19, 95%CI=2.83, 6.20,* P*<0.00001).

#### 3.4.2. Intraoperative Blood Loss

**Figure 4:**
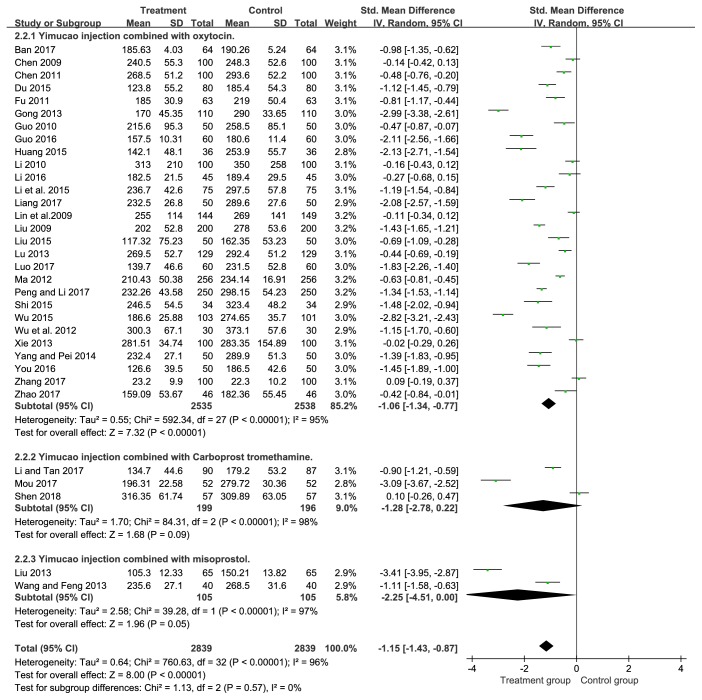
Forest plot of the meta-analysis of effects of Yimucao injection combined with western medicine versus western medicine alone for the prevention of PPH on the intraoperative blood loss.

In total, thirty-three studies [10–14, 16–18, 22–46] reported the intraoperative blood loss during cesarean section. Decreased intraoperative blood loss in response to Yimucao injection combined with western medicine (oxytocin, carboprost tromethamine, and misoprostol) group than the western medicine group alone was observed by our analysis (SMD= -1.15, 95%CI= -1.43, -0.87,* P*<0.00001). The result of this study further revealed that the treatment group performed better than the control group in improving PPH during cesarean section (Figure 4).

#### 3.4.3. Blood Loss within 2 Hours after Delivery

**Figure 5:**
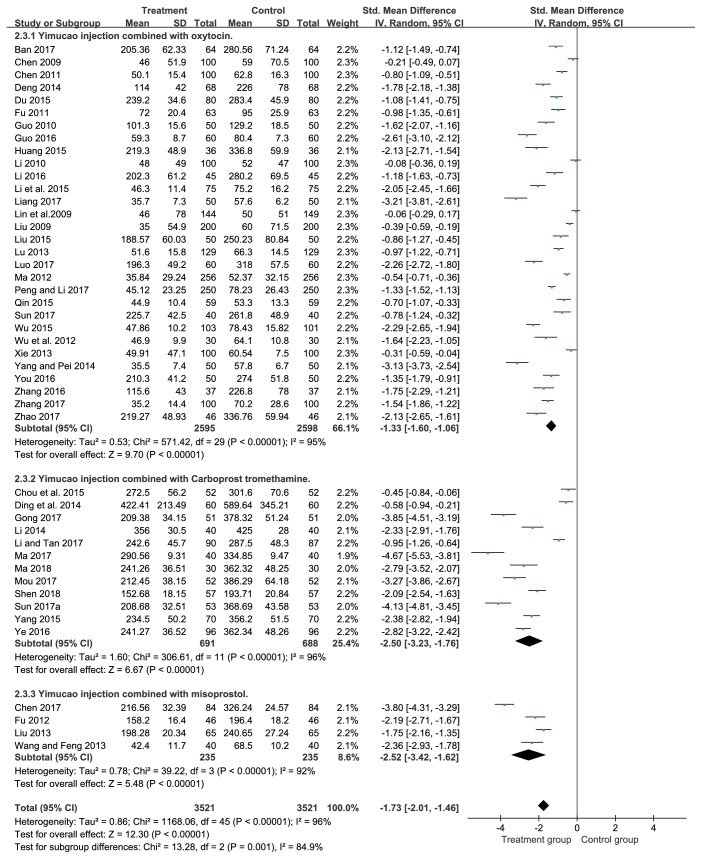
Forest plot of the meta-analysis of effects of Yimucao injection combined with western medicine versus western medicine alone for the prevention of PPH on the blood loss within 2 hours after delivery.

Forty-six RCTs [10–27, 29–39, 41–57] (n=7042) reported the outcome of blood loss within 2 hours after delivery. The incidence of blood loss in the Yimucao injection combined with western medicine group significantly decreases compared to that in the control group (SMD= -1.73, 95%CI= -2.01, -1.46,* P*<0.00001) (Figure 5).

#### 3.4.4. Blood Loss within 24 Hours after Delivery

**Figure 6:**
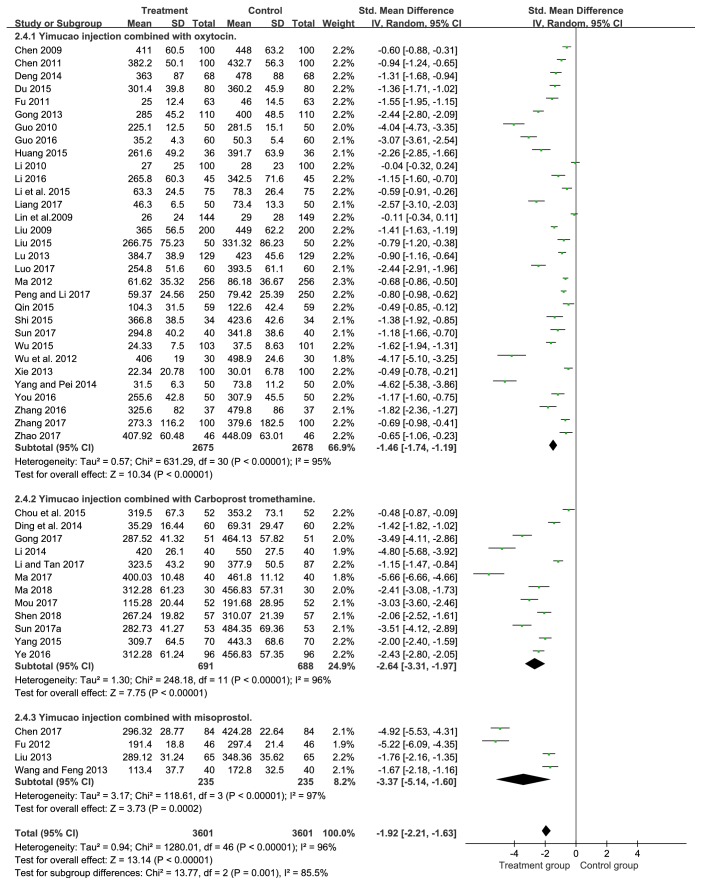
Forest plot of the meta-analysis of effects of Yimucao injection combined with western medicine versus western medicine alone for the prevention of PPH on the blood loss within 24 hours after delivery.

In our analysis, there were forty-seven studies [10–25, 27–57] providing the data of blood loss within 24 hours after delivery. Our meta-analysis showed a significant difference in blood loss within 24 hours after delivery which was witnessed between the treatment group and control group (SMD= -1.92, 95%CI= -2.21, -1.63,* P*<0.00001). The result exhibited a significant efficacy in PPH in Yimucao injection combined with western medicine compared to the western medicine alone (Figure 6).

### 3.5. Subgroup Analysis

#### 3.5.1. Yimucao Injection + Oxytocin versus Oxytocin

Participants with PPH were treated with Yimucao injection and oxytocin in the treatment group and with oxytocin in the control group. Results of subgroup analysis showed that Yimucao injection combined with oxytocin may be effectively reduces the intraoperative blood loss (SMD= -1.06, 95%CI= -1.34, -0.77,* P*<0.00001), blood loss within 2 hours (SMD= -1.33, 95%CI= -1.60, -1.06,* P*<0.00001), and blood loss within 24 hours after delivery (SMD= -1.46, 95%CI= -1.74, -1.19,* P*<0.00001) for preventing PPH compared with the oxytocin alone.

#### 3.5.2. Yimucao Injection + Carboprost Tromethamine versus Carboprost Tromethamine

Patients with PPH were treated with Yimucao injection and carboprost tromethamine in the treatment group and with carboprost tromethamine in the control group. Results of subgroup analysis demonstrated that the treatment group could significantly reduce the intraoperative blood loss (SMD= -1.28, 95%CI= -2.78, 0.22,* P*=0.09), blood loss within 2 hours (SMD= -2.50, 95%CI= -3.23, -1.76,* P*<0.00001), and blood loss within 24 hours (SMD= -2.64, 95%CI= -3.31, -1.97,* P*<0.00001) compared to the control group. Although carboprost tromethamine appeared to decrease intraoperative blood loss, the difference was not statistically significant.

#### 3.5.3. Yimucao Injection + Misoprostol versus Misoprostol

Parturient women with PPH were treated with Yimucao injection and misoprostol in the treatment group and with misoprostol in the control group. The results of subgroup analysis indicated that Yimucao injection combined with misoprostol therapy was significantly decrease compared to misoprostol alone in the intraoperative blood loss (SMD= -2.25, 95%CI= -4.51, 0.00,* P*=0.05), blood loss within 2 hours (SMD= -2.52, 95%CI= -3.42, -1.62,* P*<0.00001), and blood loss within 24 hours (SMD= -3.37, 95%CI= -5.14, -1.60,* P=*0.0002).

### 3.6. Heterogeneity and Publication Bias

**Figure 7:**
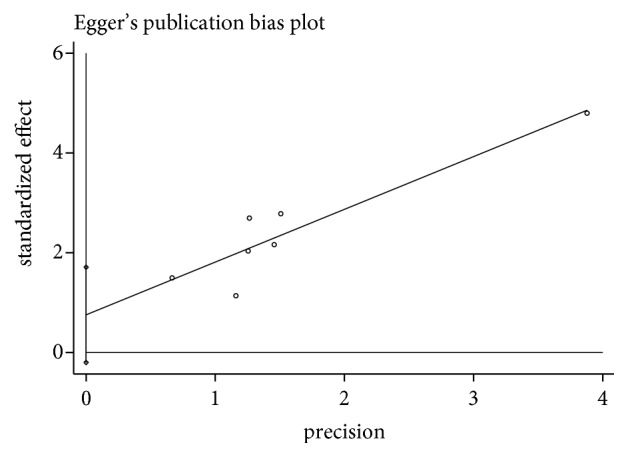
Egger's funnel plot of total effective rate.

**Figure 8:**
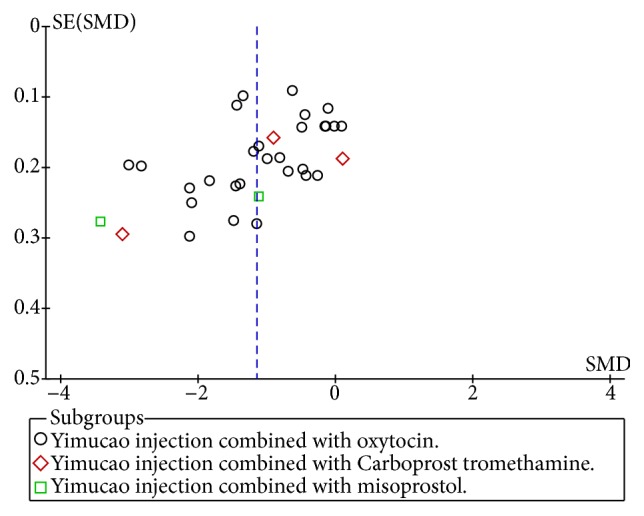
Funnel plot of intraoperative blood loss.

During the meta-analysis, we found high heterogeneity among studies. Consequently, we performed a sensitivity analysis by Egger's test of the total effective rate (Figure 7). A significant symmetry was noted for distribution in funnel plots of the intraoperative blood loss (Figure 8). These results did not demonstrate any evidence of publication bias (*P*>0.098).

### 3.7. Adverse Events

In the 48 included studies, twenty-six trials [10, 13–16, 19, 22, 23, 25–30, 32–34, 36, 41, 43–45, 49–51, 53] reported on adverse events. No adverse effects of Yimucao injection combined with western medicine group were identified in 4 trials. Only one trial reported allergic reaction in the Yimucao treatment group. However, all of these twenty-two trials reported blood pressure elevation, facial flushing, nausea, and vomiting in both the control group and treatment groups, in which 3 trials reported arrhythmia, 3 studies reported abdominal discomfort, and 2 trials reported chest discomfort. The side-effect incidence rates of the treatment group and the control group of blood pressure elevation were 1.44% and 2.23%, facial flushing 1.32% and 1.80%, nausea and vomiting 1.74% and 4.93%, abdominal discomfort 0.30% and 0.42%, chest discomfort 0.90% and 2.04%, arrhythmia 0.66% and 1.20%, and allergic reaction 0.06% and 0.00%, respectively. But all of these adverse events were not severe. No serious adverse events were reported. Symptoms of patients disappeared or significantly improved in the short term without provided treatment. In our analysis of adverse events it was suggested that the treatment group has less adverse effects than the control group (Table 2).

## 4. Discussion

### 4.1. Main Outcome

Postpartum hemorrhage (PPH) continues to be the most important cause of maternal mortality and morbidity worldwide. Therefore, in women during delivery, we should give priority to the care of the prevention and treatment of PPH. Yimucao is a Chinese herbal medicine of the genus Leonurus Lamiaceae and the main active ingredient of alkaloid. It has antithrombosis, anticoagulation, improving microcirculation, antioxygen free radical, and excited uterine smooth muscle strips and maintains the stability of intracellular calcium. Yimucao injection has been widely used in the treatment of obstetric and gynecologic disease such as abnormal menstruation, promoting gestation, postpartum hemorrhage, uterine involution, and so on. In 48 RCTs in our study, a total of 7,330 women were included in order to acquire high-quality evidence for the clinical efficacy and safety of Yimucao injection therapy in PPH. The results showed that Yimucao injection which is combined with western medicine for the treatment of PPH and significantly improved the total effective rate compares control group. Our meta-analysis shows that, compared with control group, Yimucao injection combined with western medicine has the more significant effect on the blood loss during the intraoperative, delivery hours of 2 hours and 24 hours, and it effectively reduced the amount of blood loss and risk of adverse events.

### 4.2. Subgroup Analysis

Oxytocin is one of the naturally occurring hormones, which can initiate oxytocin receptors and maintain adequate uterine tone resulting in rhythmic uterine contractions and thereby minimise blood loss [58]. Moreover, oxytocin is first-line uterotonics for prevent uterine atony and PPH during delivery [7]. The meta-analysis results showed that oxytocin combined with Yimucao injection was effective agent than oxytocin alone in reducing the blood loss during the intraoperative, 2 hours and 24 hours during cesarean section in our subgroup analysis.

Although oxytocin was found to be the most commonly used uterotonic agent for the prevention of PPH and has been reported to reduce blood loss during cesarean section [59]. However, it has a half-life of <10 min (short duration of action), negative inotropic, antiplatelet, and antidiuretic effects. The study [24] found that the effect of traditional Chinese medicine Yimucao contractile uterus was slow and lasted more than 6 hours. Thus, Yimucao injection and oxytocin have synergistic effect. Moreover, most of the studies have reported other uterotonic agents and also showed the prevention of PPH, including carboprost tromethamine and misoprostol [60–62].

Carboprost tromethamine is the synthetic 15-methyl analogue of prostaglandin F2*α*, and it was reported [5] that carboprost tromethamine for the treatment of persistent hemorrhage due to uterine atony significantly improved the effective rate by 84-96%. A study [63] reported that 237 patients were used to PPH with carboprost tromethamine, and the result showed that the improvement rate of PPH was 87.8%. This study was to compare carboprost tromethamine combined with Yimucao injection with carboprost tromethamine alone for the prevention of PPH in high-risk females undergoing cesarean delivery. Results of subgroup analysis indicated that carboprost tromethamine combined with Yimucao injection group could significantly enhance the uterine contraction for preventing PPH compared with carboprost tromethamine group.

Misoprostol is a PGE1 analogue of the strong effect on the uterus, which has been widely used for the prevention of PPH during cesarean delivery especially in developing countries [64]. Most of the studies [65–68] were suggested that misoprostol to be effective in reducing blood loss during cesarean section and it is regarded as an effective drug could substitute for other uterotonic agents. In addition, the advantages of misoprostol were inexpensive and thermostable. A few studies [34, 57] have reported the efficacy of Yimucao injection combined with misoprostol versus misoprostol alone. According to the above analysis it was demonstrated that the clinical efficacy of Yimucao injection combined with misoprostol for preventing PPH was significantly better than that of misoprostol.

This study systematically reviewed that the effect of Yimucao injection combined with western medicine for preventing PPH during cesarean section. In forty-eight RCTs in our study, a total of 26 trials reported adverse events. However, all trials did not show abnormal changes and severe adverse drug reactions during the course of treatment. Therefore, in the aspect of safety analysis, it could be judged that Yimucao injection combined with western medicine has better safety in the prevention of PPH.

### 4.3. Limitations of This Study

Although, we have comprehensive analysis and evaluate all studies, it still has limitations. First, the review includes 48 RCTs, with 7,330 women, which were published in Chinese and lacked relevant foreign experiments; the results of this study were regional and there may be publication bias. Secondly, most of the studies showed only the randomized trials, but no specific methods of random sequence generation, RCTs of allocation concealment, and blinding of outcome assessment. There were only two studies which reported double blinding. The methodological quality of many of the included RCTs was generally low and might have a high risk of bias. Third, we found that the outcome indicator of PPH in some trials was high heterogeneity. There was the difference in the doses of Yimucao injection combined with western medicine and western medicine alone in the treatment group and the control group. Furthermore, the control group was the use of different drugs in varied regimens. We considered different methods of administration in subgroup analyses, and there was still a very high heterogeneity. The source of high heterogeneity may be inconsistent with the method used to measure the amount of bleeding in clinical studies. We also found that the doses of drugs and methods of administration were different between study groups. In our meta-analysis, reporting bias is an important section. The funnel plot of the intraoperative blood loss showed symmetry and the quantitation of Egger's test with the total effective rate (*P*>0.098). The results indicated that publication bias was possibly low and partly reliable, but it could not show the whole publication biases.

## 5. Conclusions

In summary, this meta-analysis of RCTs suggested the use of Yimucao injection for the effective prevention PPH during and after cesarean section. It can effectively prevent of PPH and reduce the risk of adverse events. Subgroup analyses indicated that the clinical efficacy of Yimucao injection combined with western medicine such as oxytocin, carboprost tromethamine, and misoprostol could significantly improve PPH compared with western medicine alone. However, the methodological quality of these trials was relatively low and the significant intergroup heterogeneity in this study. Trials of larger scale or long-term, double-blind RCTs and high methodological standards are needed to provide more evidence to demonstrate the efficacy of Yimucao injection in preventing PPH during and after cesarean section.

## Figures and Tables

**Table 1:** Characteristics of included articles.

Study	Sample	Age	Intervention		Follow-up	Evaluation
	(T/C)	(T/C)	T	C		
Peng and Li, 2017 [22]	250/250	28.32/27.45	YM+OT	OT	NR	①②③④
Ban, 2017 [26]	64/64	28.45/27.56	YM+OT	OT	4d	②③④
Sun, 2017 [47]	40/40	34.98/35.41	YM+OT	OT	3d	③④
Li et al., 2015 [27]	75/75	25.2	YM+OT	OT	NR	②③④
Du, 2015 [10]	80/80	29.6/29.7	YM+OT	OT	3d	①②③④
Shi, 2015 [28]	34/34	NR	YM+OT	OT	3d	②④
Ma, 2012 [11]	256/256	24.46/24.18	YM+OT	OT	NR	②③④
Chen, 2011 [29]	100/100	29	YM+OT	OT	3d	②③④
Fu, 2011 [30]	63/63	NR	YM+OT	OT	NR	②③④
Lin et al., 2009 [12]	144/149	NR	YM+OT	OT	NR	②③④
Shen, 2018 [13]	57/57	27.95/27.86	YM+CT	CT	NR	②③④
Li and Tan, 2017 [14]	90/87	28.7/29.2	YM+CT	OT	3d	①②③④
Ye, 2016 [48]	98/98	28.89/29.56	YM+CT	CT	2d	③④
Yang, 2015 [49]	70/70	28.8/29.0	YM+CT	CT	2d	③④
Chou et al., 2015 [50]	52/52	26.6/27.3	YM+CT	OT	NR	③④
Ding et al., 2014 [15]	60/60	NR	YM+CT	OT	NR	③④
You, 2016 [31]	50/50	28	YM+OT	OT	NR	②③④
Zhang, 2016 [51]	37/37	26.3/27.9	YM+OT	OT	NR	③④
Li, 2016 [16]	45/45	24.9/24.7	YM+OT	OT	NR	②③④
Liu, 2015 [23]	50/50	28.11/27.81	YM+OT	OT	NR	①②③④
Qin, 2015 [52]	59/59	28.3/28.5	YM+OT	OT	NR	③④
Deng, 2014 [53]	68/68	28.4/29.3	YM+OT	OT	3d	③④
Xie, 2013 [32]	100/100	NR	YM+OT	OT	3d	②③④
Lu, 2013 [45]	129/129	27.5/27.9	YM+OT	OT	NR	②③④
Liu, 2009 [33]	200/200	NR	YM+OT	OT	NR	②③④
Chen, 2009 [44]	100/100	NR	YM+OT	OT	NR	②③④
Ma, 2018 [54]	30/30	29.26/28.95	YM+CT	CT	2d	③④
Wang and Feng, 2013 [34]	40/40	25.2/27.4	YM+MS+OT	MS+OT	NR	②③④
Liu, 2013 [35]	65/65	25/25.2	YM+MS	OT	NR	②③④
Li, 2010 [36]	100/100	NR	YM+OT	OT	NR	②③④
Wu et al., 2012 [37]	30/30	NR	YM+OT	OT	NR	②③④
Yang and Pei, 2014 [24]	50/50	28.09/28.16	YM+OT	OT	NR	①②③④
Liang, 2017 [17]	50/50	29.6/29.3	YM+OT	OT	NR	①②③④
Luo, 2017 [38]	60/60	29.7/29.1	YM+OT	OT	NR	②③④
Guo, 2010 [39]	50/50	27.0/30.5	YM+OT	OT	NR	②③④
Guo, 2016 [25]	60/60	30.2/30.5	YM+OT	OT	NR	①②③④
Huang, 2015 [46]	36/36	27.2	YM+OT	OT	NR	②③④
Gong, 2013 [40]	110/110	NR	YM+OT	OT	NR	②④
Wu, 2015 [41]	103/101	24.56	YM+OT	OT	NR	②③④
Zhang, 2017 [42]	100/100	22/23	YM+OT	OT	NR	②③④
Zhao, 2017 [18]	46/46	28.23/28.62	YM+OT	OT	NR	②③④
Sun, 2017 a[19]	53/53	28.09/28.21	YM+CT	CT	2d	③④
Mou, 2017 [43]	52/52	31.62/31.59	YM+CT	CT	NR	②③④
Ma, 2017 [20]	40/40	28.13/28.46	YM+CT	CT	NR	③④
Li, 2014 [55]	40/40	NR	YM+CT	CT	NR	③④
Gong, 2017 [21]	51/51	29.03/28.47	YM+CT	CT	2d	③④
Fu, 2012 [56]	46/46	26.6	YM+MS	OT	NR	③④
Chen, 2017 [57]	84/84	24.3/24.7	YM+MS	MS	3d	③④

*Note*. T: treatment group; C: control group; d: days; NR: not report; YM: Yimucao injection; OT: oxytocin; CT: carboprost tromethamine; MS: misoprostol; ①: the total effective rate; ②: intraoperative blood loss; ③: blood loss within 2 hours; ④: blood loss within 24 hours.

**Table 2:** The incidence of adverse reactions between the treatment group and control group.

	treatment group (n=1669)	control group (n=1664)
blood pressure elevation	24 (1.44%)	38 (2.23%)
facial flushing	22 (1.32%)	30 (1.80%)
nausea and vomiting	29 (1.74%)	82 (4.93%)
abdominal discomfort	5 (0.30%)	7 (0.42%)
chest discomfort	15 (0.90%)	34 (2.04%)
arrhythmia	11 (0.66%)	20 (1.20%)
allergic reaction	1 (0.06%)	0 (0.00%)

## References

[1] Henriquez D. D. C. A., Bloemenkamp K. W. M., van der Bom J. G. (2018). Management of postpartum hemorrhage: how to improve maternal outcomes?. *Journal of Thrombosis and Haemostasis*.

[2] Magann E. F., Evans S., Hutchinson M., Collins R., Lanneau G., Morrison J. C. (2005). Postpartum hemorrhage after cesarean delivery: An analysis of risk factors. *Southern Medical Journal*.

[3] Bodur S., Gun I., Ozdamar O., Babayigit M. A. (2015). Safety of uneventful cesarean section in terms of hemorrhage. *International Journal of Clinical and Experimental Medicine*.

[4] Luo A., Mao P. (2015). Late postpartum hemorrhage due to placental and fetal membrane residuals: experience of two cases. *Clinical and Experimental Obstetrics and Gynecology*.

[5] Bai J., Sun Q., Zhai H. (2013). A comparison of oxytocin and carboprost tromethamine in the prevention of postpartum hemorrhage in high-risk patients undergoing cesarean delivery. *Experimental and Therapeutic Medicine*.

[6] Afolabi E. O., Kuti O., Orji E. O., Ogunniyi S. O. (2010). Oral misoprostol versus intramuscular oxytocin in the active management of the third stage of labour. *Singapore Medical Journal*.

[7] Tunçalp Ö., Souza J. P., Gülmezoglu M. (2013). New WHO recommendations on prevention and treatment of postpartum hemorrhage. *International Journal of Gynecology & Obstetrics*.

[8] Pantoja T., Abalos E., Chapman E., Vera C., Serrano V. P. (2016). Oxytocin for preventing postpartum haemorrhage (PPH) in non-facility birth settings. *Cochrane Database of Systematic Reviews*.

[9] Chen Z., Wu J. B., Liao X. J., Yang W., Song K. (2010). Development and validation of an UPLC-DAD-MS method for the determination of leonurine in Chinese motherwort (Leonurus japonicus). *Journal of Chromatographic Science (JCS)*.

[10] Du W. Y. (2015). Curative effects and clinical safety of leonurus injection combined with oxytocin in prevention of postpartum hemorrhage after secarean section. *Chinese Journal of Family Planning*.

[11] Ma B. (2012). Analysis to the impact of combined use of motherwort injection with oxytocin on hemorrhage in cesarean section and postoperative stage. *Journal of Traditional Chinese Medicine*.

[12] Lin J. H., Lin Q. D., Liu X. H. (2009). Multi-center study of motherwort injection to prevent postpartum hemorrhage after caesarian section. *Chinese Journal of Obstetrics and Gynecology*.

[13] Shen J. P. (2018). Effect of motherwort injection combined with prostaglandin trometamol on prevention of cesarean section postpartum hemorrhage in high -risk pregnant women. *Chinese Journal of Family Planning Gynecotokology*.

[14] Li Y. M., Tan B. J. (2017). Clinical observation of leonurus artemisia injection combined with carboprost tromethamine for prevent-ing postpartum hemorrhage after cesarean section. *China Pharmacy*.

[15] Ding X. Y., Feng T., Geng H., Liu F., Ma A. R. (2014). Clinical study of carboprost tromethamine combined with motherwort injection in prevention of high-risk ce-sarean section hemorrhage. *Chinese Journal of Primary Medicine and Pharmacy*.

[16] Li J. M., Wang X. Z. (2016). Study on the efficacy of carbetocin combined with yimucao injection in cesarean section. *Maternal and Child Health Care of China*.

[17] Liang X. X. (2017). Research of synergistic effect by leonurus heterophyllus injection for oxytocin in the treatment of postpartum hemorrhage in cesarean section uterine inertia. *China Practical Medical*.

[18] Zhao D. H. (2017). The clinical effect of oxytocin combined with Yimucao injection in preventing postpartum hemorrhage in cesarean section. *Guide of China Medicine*.

[19] Sun L. (2017). Effects of yimucao injection and oxytocin on hemorrhage and blood coagulation index after cesarean section. *China Health Care & Nutrition*.

[20] Ma W. J. (2017). Clinical study on prevention of postpartum hemorrhage due to cesarean section by intravenous injection of pretreatment with cardioprost and tandromeuse injection. *Modern Diagnosis and Treatment*.

[21] Gong H. Y., Xia M. (2017). Effect observation of leonurus heterophyllus injection combined with carboprost tromethamine in the prevention of caesarean postpartum hemorrhage. *IMHGN*.

[22] Peng L. Q., Li L. (2017). Yimucao Injection combined with oxytocin in preventing postpartum hemorrhage after cesarean section. *Drug Evaluation and Research*.

[23] Liu S. H. (2015). Clinical efficacy and safety of yimucao injection combined with oxytocin in preventing postpartum hemorrhage in cesarean section. *Journal of North Pharmacy*.

[24] Yang X. J., Pei Y. S. (2014). Observation on the application of yimucao injection and oxytocin to bleeding in and after the surgery of cesarean section. *Western journal of chinese medicine*.

[25] Guo M. X. (2016). The leonurus injection combined with oxytocin in the treatment of intraoperative and postoperative hemorrhage in cesarean section. *China Continuing Medical Education*.

[26] Ban F. Q. (2017). Clinical effect observation of Motherwort injection combined with Oxytocin in prevention of postpartum hemorrhage aftercesarean section. *Medical Journal of Chinese People's Health*.

[27] Li S. Y., Liu X. C., Zhang Y. M., Zhang G. Y., Cao S. Y. (2015). Leonurus japonicus injection in combined with oxytocin on postpartum hemorrhage after cesarean section. *Changchun University of Chinese Medicine*.

[28] Shi R. P. (2015). Effect of leonurus heterophyllus injection combined with oxytocin on preventing patients from hemorrhage after cesarean section. *Acta Medicinae Sinica*.

[29] Chen H. (2011). Motherwort injection combined with oxytocin in the prevention of pre-and post-operative hemorrhage in cesarean section: A clinical observation. *Journal of Huaihai Medicine*.

[30] Fu Y. F. (2011). Motherwort combined with oxytocin in the prevention of postpartum hemorrhage and the acceleration of uterine restoration. *IMHGN*.

[31] You A. P. (2016). Effect of Yimucao injection and oxytocin on prevention of intraoperative and postpartum hemorrhage in cesarean section. *The World Clinical Medicine*.

[32] Xie X. Q., Liu J. (2013). Treatment of 100 cases of cesarean section hemorrhage with Yimucao injection and oxytocin. *Chinese Medicine Modern Distance Education of China*.

[33] Liu S. J. (2009). Clinical observation on prevention of hemorrhage after cesarean section with yimucao injection. *Maternal and Child Health Care of China*.

[34] Wang M. Y., Feng R. H. (2013). Efficacy of misoprostol combined with Yimucao for preventiny postoperative bleeding of cesarean section. *China Modern Doctor*.

[35] Liu Z. F. (2013). Clinical observation on prevention of postoperative hemorrhage after cesarean section with yimucao injection combined with misoprostol. *Hebei Journal of Traditional Chinese Medicine*.

[36] Li H. M., Zang L. L. (2010). Observation of curative effect on motherwort injection, misoprostol tablets and oxytocin in conjunction with the prevention of postpartum hemorrhage. *Chinese Journal of Medicinal Guide*.

[37] Wu X. M., Li Y. P., Hu C. X., Jin S. (2012). Application of yimucao injection in cesarean section of twin pregnancy. *Journal of Obstetrics and Gynaecology*.

[38] Luo Y. F. (2017). Effect of Yimucao injection and oxytocin on prevention of hemorrhage after cesarean section. *Nei Mongol Journal of Traditional Chinese Medicine*.

[39] Guo J. H. (2010). Effects of yimucao Injection and oxytocin on postpartum hemorrhage and estrogen levels. *China Journal of Traditional Chinese Medicine and Pharmacy*.

[40] Gong J. (2013). Therapeutic effect of yimucao injection combined with oxytocin on prevention of postpartum hemorrhage in selective cesarean section. *Chinese Journal of Modern Drug Application*.

[41] Wu H. (2015). Effect of Yimucao injection combined with oxytotin on intraoperatve and postoperative bleeding in cesarean section. *Modern Journal of Integrated Traditional Chinese and Western Medicine*.

[42] Zhang Y. J., Zhang J. W. (2017). Effect of Yimucao injection and oxytocin on cesarean section. *Shenzhen Journal of Integrated Traditional Chinese and Western Medicine*.

[43] Mou Y. Y. (2017). Effect of Yimucao injection combined with cardoprost tromethamine on prevention of hemorrhoids caused by hysterocolic force in cesarean section. *Chinese Primary Health Care*.

[44] Chen T. M., Zhu J. H., Yuan H. L. (2009). Clinical observation on prevention of hemorrhage after cesarean section by combined use of yimucao injection and oxytocin. *Journal of Clinical Research*.

[45] Lu L. P. (2013). Analysis on effect of leonurus heterophyllus injection combined oxytocin in the prevention of postpartum hemorrhage after cesarean delivery. *Chinese Journal of Modern Drug Application*.

[46] Huang L. J. (2015). Clinical observation on prevention of postpartum hemorrhage by yimucao Injection combined with oxytocin in cesarean section. *J. hebei. Nat. Sci*.

[47] Sun K. J. (2017). Therapeutic effect of yimucao injection combined with cardoprost tromethamine on preventing hemorrhoids caused by hysterocolic force. *Journal of North Pharmacy*.

[48] Ye X. C. (2016). Effect of Leonurus heterophyllus injection combined with carboprost tromethamine on preventing haemorrhage after cesarean section and its significance on coagulation. *China Modern Medicine*.

[49] Yang X. S., Xiong L. (2015). Curative efficacy of yimucao injection combined with carboprost tromethamine in preventing cesarean section postpartum hemorrhage and on FIB and D-dimer levels. *Chinese Journal of Experimental Traditional Medical Formulae*.

[50] Chou Q. M., Du Z. M., Dai S. (2015). Therapeutic evaluation of postpartum hemorrhage by carboprost tromethamine and motherwort injection in preeclampsia. *Strait Pharmaceutical Journal*.

[51] Zhang H. (2016). Effect of Yimucao combined with oxytocin on postpartum hemostasis. *Journal of Clinical Medical Literature*.

[52] Qin X. F. (2015). Clinical efficacy of yimucao injection combined with oxytocin in the treatment of postpartum hemorrhage. *Chinese Journal of Clinical Rational Drug Use*.

[53] Deng X. Q. (2014). Therapeutic effect of yimucao injection combined with oxytocin on prevention of postpartum hemorrhage. *Modern Journal of Integrated Traditional Chinese and Western Medicine*.

[54] Ma G. Q. (2018). Analysis of Curative effect of yimucao injection combined with cardoprost tromethamine on prevention of postpartum hemorrhage in cesarean section. *Women's Health Research*.

[55] Li J. L. (2014). Effect of uterine body injection of carboprost tromethamine injection combined with motherwort injection in the prevention of cesarean section's postpartum hemorrhage. *Chinese Journal of New Clinical Medicine*.

[56] Fu C. H., Shi P. J. (2012). Effect of misoprostol combined with yimucao injection in prevention and treatment of postpartum hemorrhage in cesarean section. *Chinese Community Doctors*.

[57] Chen H. Z., Liu Q. (2017). Standardized measures to assist misoprostol in combination with intrauterine injection of Yimucao in preventing cesarean section hemorrhage during pregnancy-induced hypertension and its effect on coagulation system. *Journal of Preventive Medicine of Chinese People's Liberation Army*.

[58] Meshykhi L. S., Nel M. R., Lucas D. N. (2016). The role of carbetocin in the prevention and management of postpartum haemorrhage. *International Journal of Obstetric Anesthesia*.

[59] Roach M., Abramovici A., Tita A. N. (2013). Dose and duration of oxytocin to prevent postpartum hemorrhage: A review. *American Journal of Perinatology*.

[60] Su L. L., Chong Y. S., Samuel M. (2012). Carbetocin for preventing postpartum haemorrhage. *Cochrane Database Syst Rev*.

[61] Cordovani D., Balki M., Farine D., Seaward G., Carvalho J. C. A. (2012). Carbetocin at elective Cesarean delivery: A randomized controlled trial to determine the effective dose. *Canadian Journal of Anesthesia*.

[62] Than K. K., Mohamed Y., Oliver V. (2017). Prevention of postpartum haemorrhage by community-based auxiliary midwives in hard-to-reach areas of Myanmar: A qualitative inquiry into acceptability and feasibility of task shifting. *BMC Pregnancy and Childbirth*.

[63] Oleen M. A., Mariano J. P. (1990). Controlling refractory atonic postpartum hemorrhage with Hemabate sterile solution. *American Journal of Obstetrics & Gynecology*.

[64] Othman E. R., Fayez M. F., El Aal D. E. M. A., El-Dine Mohamed H. S., Abbas A. M., Ali M. K. (2016). Sublingual misoprostol versus intravenous oxytocin in reducing bleeding during and after cesarean delivery: A randomized clinical trial. *Taiwanese Journal of Obstetrics and Gynecology*.

[65] Gülmezoglu A. M., Villar J., Ngoc N. T. (2001). WHO multicentre randomised trial of misoprostol in the management of the third stage of labour. *The Lancet*.

[66] Miller S., Lester F., Hensleigh P. (2004). Prevention and treatment of postpartum hemorrhage: new advances for low-resource settings. *Journal of Midwifery & Women’s Health*.

[67] Nielsen B. B., Høj L., Hvidman L. E., Nielsen J., Cardoso P., Aaby P. (2006). Reduced post-partum bleeding after treatment with sublingual misoprostol: A randomized double-blind clinical study in a developing country (secondary publication). *Ugeskrift for Læger*.

[68] Baskett T. F., Persad V. L., Clough H. J., Young D. C. (2007). Misoprostol versus oxytocin for the reduction of postpartum blood loss. *International Journal of Gynecology and Obstetrics*.

